# Serologic Evidence for Influenza C and D Virus among Ruminants and Camelids, Africa, 1991–2015

**DOI:** 10.3201/eid2309.170342

**Published:** 2017-09

**Authors:** Elias Salem, Elizabeth A.J. Cook, Hicham Ait Lbacha, Justine Oliva, Félix Awoume, Gilbert L. Aplogan, Emmanuel Couacy Hymann, Dishon Muloi, Sharon L. Deem, Said Alali, Zaid Zouagui, Eric M. Fèvre, Gilles Meyer, Mariette F. Ducatez

**Affiliations:** Interactions Hôtes Agents Pathogènes, Université de Toulouse, INRA, ENVT, Toulouse, France (E. Salem, J. Oliva, G. Meyer, M.F. Ducatez);; International Livestock Research Institute, Nairobi, Kenya (E.A.J. Cook, D. Muloi, E.M. Fèvre);; Institut Agronomique et Vétérinaire Hassan II, Rabat, Morocco (H.A. Lbacha, S. Alali, Z. Zouagui);; Laboratoire Vétérinaire de Lomé, Lomé, Togo (F. Awoume);; Laboratoire de Diagnostic Vétérinaire et de Sérosurveillance, Parakou, Benin (G.L. Aplogan);; LANADA Central Laboratory for Animal Diseases, Bingerville, Côte d’Ivoire (E.C. Hymann);; University of Edinburgh Centre for Immunity, Infection, and Evolution, Edinburgh, Scotland, UK (D. Muloi);; St. Louis Zoo Institute for Conservation Medicine, St. Louis, Missouri, USA (S.L. Deem);; University of Liverpool Institute of Infection and Global Health, England, UK (E.M. Fèvre)

**Keywords:** influenza virus, influenza, influenza C virus, influenza D virus, bovine, cattle, dromedary, camel, ruminant, camelid, swine, goats, sheep, Kenya, Morocco, Benin, Togol, Côte d’Ivoire, Africa, serology

## Abstract

Influenza D virus has been identified in America, Europe, and Asia. We detected influenza D virus antibodies in cattle and small ruminants from North (Morocco) and West (Togo and Benin) Africa. Dromedary camels in Kenya harbored influenza C or D virus antibodies, indicating a potential new host for these viruses.

Influenza D virus (IDV) was recently discovered in the United States in a pig with influenza-like symptoms (*1*). So far, IDV or IDV antibodies have been detected in the United States, Mexico, France, Italy, China, and Japan, in healthy or sick cattle and pigs that had respiratory signs (*1**–**6*) (Figure 1). The pathogenesis and transmission of this virus are not fully understood, but recent experimental infection of calves showed that IDV can cause moderate respiratory disease (*7*) and that the virus is related to the bovine respiratory disease complex (*2*), which is a disease with very large economic costs and public health impact. The ability of IDV to replicate in ferrets, the animal model of choice for studying influenza virus in humans (*1*), and in guinea pigs (*8*) indicates that IDV might have a wider host range than currently expected and that humans may be susceptible to infection. In addition to swine and cattle, anti-IDV antibodies have been detected in goats and sheep (*9*). We conducted a study to assess the putative IDV circulation in Africa. 

**Figure 1:**
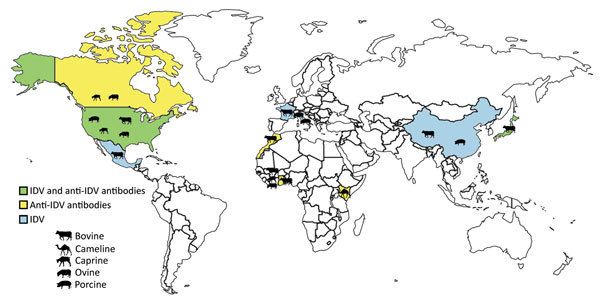
Locations where IDV or IDV antibodies had been detected as of April 2017. Species from which virus or antibodies were detected are indicated. IDV, influenza D virus.

## The Study

During 1991–2015, a total of 2,083 serum samples were collected from cattle, swine, small ruminants, and dromedary camels in Morocco (n = 200), Togo (n = 540), Côte d’Ivoire (n = 203), Benin (n = 308), and Kenya (n = 1,231) (Table 1). We screened these samples by hemagglutination inhibition (HI) and microneutralization (MN) assays as described in the World Health Organization Manual for the Laboratory Diagnosis and Virological Surveillance of Influenza (*10*) (Technical Appendix).

**Table 1:** Number of serum samples collected per country in Africa during 1991–2015 and species and collection periods and influenza D virus seroprevalence*

Country	**Species**				
	Cattle	Swine	Sheep	Goats	Camels
Benin					
% Positive	1.9	ND	0	0	ND
No. samples	207 [1]	ND	67	34	ND
Years	2012, 2014	ND	2013–2014	2013–2014	ND
Togo					
% Positive	10.4	ND	2.2	1.4	ND
No. samples	201 [10]	ND	135 [2]	205 [0]	ND
Years	2009, 2015	ND	2013	2013	ND
Côte d’Ivoire					
% Positive	0	0	ND	ND	ND
No. samples	100	103	ND	ND	ND
Years	1991–2013	2013	ND	ND	ND
Morocco					
% Positive	35%	ND	ND	ND	ND
No. samples	200 [32]	ND	ND	ND	ND
Years	2012–2015	ND	ND	ND	ND
Kenya					
% Positive	0	ND	ND	ND	99†
No. samples	938	ND	ND	ND	293 [287]
Years	2010–2012	ND	ND	ND	2015

*A total of 2,083 serum samples were collected. Seroprevalence defined by HI titers ≥10 against D/bovine/Nebraska/9-5/2012. Numbers in brackets indicate animals with HI titers ≥40. HI, hemagglutina­tion inhibition; ND, not done. †No preadsorption on influenza C virus cross-reactivity likely.

**Figure 2:**
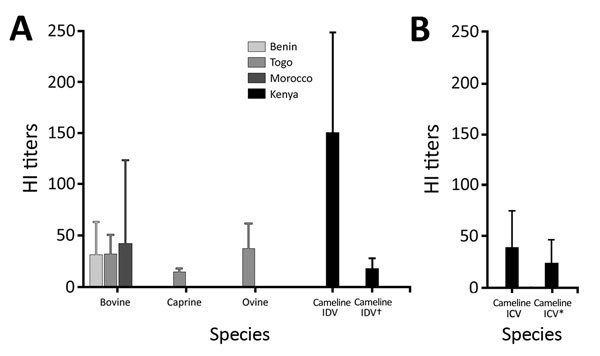
HI titers for ICV and IDV in serum samples from animals in Africa, by country. A) Titers against IDV by using D/bovine/Nebraska/9-5/2012 as antigen. B) Titers against ICV by using C/Victoria/1/2011 as antigen. Histograms represent mean HI titers per country and species as indicated on the *x*-axis. Error bars indicate SEM.* Post-IDV adsorption; †post-ICV adsorption. HI, hemagglutination inhibition; ICV, influenza C virus; IDV, influenza D virus.

Our results show that IDV has been circulating in North and West Africa since at least 2012, as shown by the antibodies detected in cattle in Morocco (from 2012 to 2015), cattle in Benin and Togo (as of 2014), and small ruminants in Togo (as of 2013) (Table 1; Figure 1). HI titers were low in ruminants, ranging 10–80 in West Africa and 10–640 in Morocco; geometric mean titers ranged 13–42 (Figure 2; HI antigen was D/bovine/Nebraska/9-5/2012). More recently, serum samples were more likely to be positive for IDV antibodies, as shown by a higher seroprevalence over time in cattle samples from Morocco and Togo (23%, 41%, and 42% seroprevalence in Morocco in 2013, 2014, and 2015, respectively; 0 and 21% seroprevalence in Togo in 2009 and 2015, respectively). None of the samples from swine or cattle in Côte d’Ivoire or small ruminants in Benin were IDV antibody–positive (Table 1; Figure 1).

To confirm our results, we tested samples from the Moroccan cohort (n = 200 cattle samples; Table 1) by using MN and HI with D/bovine/France/5920/2014 as antigen. These assays were in substantial agreement with a Cohen kappa coefficient (κ) of 0.647 (95% CI 0.541–0.753); 68% of the MN-positive samples were also positive by HI (Table 2). The agreement between HI assays with D/bovine/Nebraska/9-5/2012 and D/bovine/France/5920/2014 showed even more substantial agreement (κ = 0.796, 95% CI 0.709–0.883). All samples from Benin and Togo that were positive by HI using D/bovine/Nebraska/9-5/2012 were tested with D/bovine/France/5920/2014 and showed consistent positive results.

**Table 2:** Comparison of HI and MN assay results for influenza D virus in 200 cattle serum samples from Morocco*

HI assay	MN assay		Total no.
No. positive	No. negative
No. positive	66	4	70
No. negative	31	99	130
Total no.	97	103	200
Comparision†	Sensitivity, 68% (95% CI 57.8%–77.2%)	Specificity, 96% (95% CI 90.4%–98.9%)	

*By using D/bovine/France/5920/2014 as antigen. Titers ≥10 were considered positive. HI, hemagglutination inhibition; MN, microneutralization. †For HI as compared with MN.

We then assessed IDV circulation in Kenya. None of the cattle serum samples were positive (Table 1). We first tested the 2015 camel samples from Kenya by using HI with both IDV antigens; testing with D/bovine/Nebraska/9-5/2012 showed 99% seroprevalence and with D/bovine/France/5920/2014 100% seroprevalence (Table 1; data not shown). HI titers were higher than those observed with ruminant samples from North and West Africa (20<HI titers<640, geometric mean titer = 150; Figure 2). When tested by using C/Victoria/1/11, the seroprevalence was 94% (10<HI titers<320, geometric mean titer = 38), suggesting ICV/IDV cross-reactivity. The samples were therefore adsorbed on 4 hemagglutination units of C/Victoria/1/11 and hemadsorbed before being retested in HI with D/bovine/Nebraska/9-5/2012 and vice versa (all 293 samples were retested for IDV antibodies after preadsorption with ICV; 85 samples were preadsorbed on IDV and retested for ICV antibodies). Seroprevalences were 8.2% for IDV and 10.6% for ICV. All but 1 of the samples that were positive for IDV antibodies without ICV preadsorption lost >2 log_2_ (>4-fold decrease in titer) in HI titer once adsorbed on ICV, suggesting these samples had anti-ICV rather than anti-IDV antibodies. The picture was less clear for the reverse experiment: 11% of the IDV preadsorbed samples lost >2 log_2_ in titer (false ICV antibodies positive); 9% stayed within the 4-fold range (true positives); and the initial ICV antibody titer of the remaining 80% was too low (HI titers of 10 or 20) to determine a status post-IDV adsorption. Taken together, our serology results on camel samples show that almost all the animals had either anti-IDV or ICV antibodies, that there is cross-reactivity in camels between the 2 viruses, and that 9% of the tested samples had anti-ICV antibodies. Camels could therefore be a newly discovered host for ICV, and possibly for IDV. IDV/ICV cross-reactivity was ruled out for bovine samples after a cohort from France was preadsorbed the same way and retested in IDV HI without any change in HI titers (data not shown). Detection of antibodies against IDV in ruminants in Africa raises the question of the virus origin and transmission route. Although the virus has already been reported on 3 continents, the ruminant import/export from/to North and West Africa is limited (e.g., 21,000 cattle imported from Europe to Morocco, no exportations reported; no import or export of cattle reported to or from Togo or Benin; data for North and West Africa, 2013 [*11*]). Seroprevalences we calculated may also be underestimated because our HI assay was less sensitive than our MN assay (Table 2); numerous freezing and thawing cycles may have altered the samples; and our low titers in ruminants might have been caused by the circulation of a different IDV lineage in Africa or to the unique structure of camel antibodies, which are devoid of light chains and CH1 domain.

Although influenza A viruses are known to have nonhuman maintenance hosts, little is known on the host tropism of IDV and ICV. So far cattle, swine, sheep, goats, guinea pigs, and ferrets have been reported to be susceptible to IDV infection (*1**,**6**,**8**,**9*) and swine, dogs, and humans to ICV infections (*12*,*13*). Many aspects of camel health had not been studied before the emergence of Middle East respiratory syndrome coronavirus (*14*), but camels had been reported susceptible to influenza A(H1N1) on 1 occasion (*15*). Our data suggest that ICV and IDV have a wide host tropism and that further investigations on host tropism and on ICV and IDV circulation in camels are warranted.

## Conclusions

Our results show that IDV is circulating in Africa. This virus has a wide host tropism because cattle, swine, small ruminants, and likely dromedary camels seem susceptible to IDV infection. In addition, we show that camels in Kenya are positive for ICV antibodies, suggesting that this virus also has a wider host range than previously thought. Further studies are warranted to clarify the cross-reactivity of the 2 viruses in serologic assays, to determine which IDV lineages circulate in Africa, and to assess whether ICV alone or both ICV and IDV may infect camels.

Technical Appendix Serologic assay methods used for consideration of cross-reactivity between influenza viruses D and C in animals in Africa.
