# Supplementary material for: Serologic Evidence for Influenza C and D Virus among Ruminants and Camelids, Africa, 1991–2015
Source: Emerg Infect Dis. 2017 Sep;23(9):1556–9. doi: 10.3201/eid2309.170342 (PMC5572875; doi:10.3201/eid2309.170342)
Supplement: Technical Appendix — Serologic assay methods used for consideration of cross-reactivity between influenza viruses D and C in animals in Africa. [file 17-0342-Techapp-s1.pdf]

Article DOI: https://doi.org/10.3201/eid2309.170342

Serologic Evidence for Influenza C and D Virus among Ruminants and Camelids, Africa, 1991–2015

Technical Appendix

Material and Methods

Sera

In Morocco, sera were collected in 16 provinces (in alphabetic order, with the number of samples collected and the collection month and year per province indicated in brackets): Agadir (n = 15, April 2014), Ain Aouda (n = 12, October 2015), Beni Mellal (n = 21, September 2015), Errachidia (n = 4, October 2013), Fes (n = 6, October 2015), Gharb (n = 22, November 2013), Haouz (n = 16, September 2015), Khenifra (n = 2, September 2013), Mrirt (n = 13, November 2013), Oriental (n = 5, May 2012), Oulmes (n = 7, November 2013), Settat (n = 43, September 2015), Tanger (n = 12, October 2015), Taza (n = 10, January 2014), and Zair (n = 12, February 2014). In Benin, all ruminant sera originated from the Northern part of the country and were collected in 2012 and 2014 for the bovine sera, in 2013–2014 for the small ruminant sera. In Togo, all sera originated from the Lomé area (Southern part of the country) and were collected in 2009 and 2015 for the bovine sera, and in 2013 for the small ruminant sera. In Côte d’Ivoire, all bovine sera were collected from the northeastern part of the country (Bouaké, Panya, and Boundiali, in 1991–1992) and from the southwestern part of the country (Bingerville and Grand Bassam, in 2013–2014) for the swine sera. In Kenya, dromedary camels were sampled before slaughter in the central camel slaughterhouse in Nairobi, Kenya. All were therefore of slaughter age and were geographically representative of all camel keeping regions of the country. The Kenyan cattle was sampled from 2010 through 2012 in the Western part of the country (close to Lake Victoria, GPS coordinates: 0.084<latitude<0.740; 33.995<longitude<34.527).

Hemagglutination inhibition (HI) and microneutralisation (MN) assays

Serologic assays were performed as previously described (*1*). The sera were all treated with receptor destroying enzyme (RDE) (Seika, Japan) and hemadsorbed on packed horse red blood cells (red blood cell). Four hemagglutination units of D/bovine/Nebraska/9–5/2012

(kindly provided by Dr. Ben Hause) or D/bovine/France/5920/2014 and 1% horse red blood cell were used for HI assays. To check for influenza C virus putative cross-reactivity, we used C/Victoria/1/2011 (kindly provided by Dr. Richard Webby) and 1% chicken red blood cell. The MN assays were carried out on swine testis cells (ATCC), using 100 tissue culture infectious doses 50 per well and 5 days' incubation at 37°C and 5% CO_2 without TPCK trypsin. HI and MN titers were considered positive when ≥10.

Positive IDV reference serum was produced in-house by inoculating rabbits subcutaneously with purified D/bovine/Nebraska/9–2/2012. We also used IDV positive French cattle serum (from an experimental infection) and BEI Resources (NIAID) chicken antiserum C/Taylor/1233/47. The reference sera were RDE treated and hemadsorbed. When adsorbing both (i) the IDV reference antiserum on ICV and (ii) the ICV reference antiserum on IDV, and retesting for the respective antigens, we lost 1 log2 in titer, suggesting a 2-fold difference in HI titer is the "normal" loss of antibody due to the additional treatment of the sera. We therefore considered a 2 log2 decrease in titer as the threshold to consider cross-reactivity between ICV and IDV.

Reference

1. World Health Organization. Manual for the laboratory diagnosis and virological surveillance of influenza. Geneva: The Organization; 2011.
